# Development of Machine Learning Models to Predict Probabilities and Types of Stroke at Prehospital Stage: the Japan Urgent Stroke Triage Score Using Machine Learning (JUST-ML)

**DOI:** 10.1007/s12975-021-00937-x

**Published:** 2021-08-14

**Authors:** Kazutaka Uchida, Junichi Kouno, Shinichi Yoshimura, Norito Kinjo, Fumihiro Sakakibara, Hayato Araki, Takeshi Morimoto

**Affiliations:** 1 Department of Neurosurgery, Hyogo College of Medicine, Nishinomiya, Japan; 2 Department of Clinical Epidemiology, Hyogo College of Medicine, 1-1 Mukogawa, Nishinomiya, Hyogo 663-8501 Japan; 3 Department of Neurosurgery, Araki Neurosurgical Hospital, Hiroshima, Japan

**Keywords:** Machine learning, Prehospital, Triage, Large vessel occlusion, Intracranial hemorrhage, Subarachnoid hemorrhage

## Abstract

In conjunction with recent advancements in machine learning (ML), such technologies have been applied in various fields owing to their high predictive performance. We tried to develop prehospital stroke scale with ML. We conducted multi-center retrospective and prospective cohort study. The training cohort had eight centers in Japan from June 2015 to March 2018, and the test cohort had 13 centers from April 2019 to March 2020. We use the three different ML algorithms (logistic regression, random forests, XGBoost) to develop models. Main outcomes were large vessel occlusion (LVO), intracranial hemorrhage (ICH), subarachnoid hemorrhage (SAH), and cerebral infarction (CI) other than LVO. The predictive abilities were validated in the test cohort with accuracy, positive predictive value, sensitivity, specificity, area under the receiver operating characteristic curve (AUC), and *F* score. The training cohort included 3178 patients with 337 LVO, 487 ICH, 131 SAH, and 676 CI cases, and the test cohort included 3127 patients with 183 LVO, 372 ICH, 90 SAH, and 577 CI cases. The overall accuracies were 0.65, and the positive predictive values, sensitivities, specificities, AUCs, and *F* scores were stable in the test cohort. The classification abilities were also fair for all ML models. The AUCs for LVO of logistic regression, random forests, and XGBoost were 0.89, 0.89, and 0.88, respectively, in the test cohort, and these values were higher than the previously reported prediction models for LVO. The ML models developed to predict the probability and types of stroke at the prehospital stage had superior predictive abilities.

## Introduction

Timely intervention with thrombectomy is crucial in patients with acute stroke due to large vessel occlusion (LVO). The American Heart Association guidelines recommend thrombectomy within 6–24 h of LVO onset [1–3]. Therefore, time constraint is an important factor in implementing treatment strategies and successfully saving lives. It is also strongly recommended that patients suspected with LVO should be transported to thrombectomy-performing facilities at the earliest. Several prehospital LVO prediction scales have been developed to challenge this time constraint issue [4–7]. The positive predictive values of these scales were up to 32% [8], with substantial areas to improve them.

There are other types of acute stroke, including intracranial hemorrhage (ICH) and subarachnoid hemorrhage (SAH), and such conditions are also critical for immediate intervention. Therefore, prediction scales to predict LVO alone might not be able to shorten the transportation time for strokes other than LVO. We developed the Japan Urgent Stroke Triage (JUST) score and the 7-Item Japan Urgent Stroke Triage (JUST-7) score, which could predict any stroke and differentiate among LVO, ICH, SAH, and cerebral infarction (CI) other than LVO in patients suspected of having acute stroke, by emergency medical services (EMS) [9, 10]. Because the JUST and JUST-7 scores calculate the individual probability of each type of stroke, EMS need to determine which stroke type should be prioritized if several strokes are predicted with similar probabilities.

Machine learning (ML) had been applied in various fields owing to their high predictive performance, including stroke management [11, 12]. Thus, we applied ML methods to the JUST score to develop models to calculate the predictive probabilities of each type of stroke at the same time.

## Methods

### Study Design and Patient Population

We conducted a retrospective and prospective multi-center cohort study to develop ML models to predict the four types of stroke. The data consisted of two cohorts for training and testing the ML model. The training cohort comprised a retrospective and prospective cohort study conducted at eight centers from June 1, 2015, to March 31, 2018, in three cities of Japan. This cohort was utilized to develop the previous version of the JUST score [9, 10]. The test cohort was a prospective cohort study conducted at 13 centers from April 1, 2019, to March 31, 2020, in another city of Japan.

The inclusion criteria of two cohorts were consecutive patients who were suspected of having a stroke by the EMS and were transported to the participating centers. The participating centers covered the corresponding regions (Nishinomiya, Hirosaki, and Kobe for the training cohort and Hiroshima for the test cohort), and all suspected patients were transported to one of the participating centers. There were no age limitations. The exclusion criteria were those who were suspected of having other conditions, such as cardiovascular diseases, but were finally diagnosed as having any type of stroke. Patients with missing data for potential variables were also excluded from the analysis.

All included patients underwent diagnostic assessment with either computed tomography (CT) or magnetic resonance imaging (MRI) at the centers to determine the outcomes. The Institutional Review Boards of all participating centers approved the study protocol. Written informed consent was waived for this study because we used information obtained during routine clinical practice, and the Institutional Review Boards approved this waiver in accordance with the Ethical Guidelines for Medical and Health Research Involving Human Subjects in Japan.

### Selected Variables

Based on a previous report [9], we collected information on the following variables: (1) age, (2) sex, (3) smoking status, (4) history of cerebral infarction, (5) sudden onset of symptoms, (6) improvement after symptom onset, (7) progression after symptom onset, (8) headache, (9) dizziness, (10) convulsion, (11) nausea or vomiting, (12) systolic blood pressure ≥ 165 mmHg, (13) diastolic blood pressure ≥ 95 mmHg, (14) arrhythmia, (15) disturbance of consciousness, (16) aphasia, (17) dysarthria, (18) conjugate deviation, (19) unilateral spatial neglect, (20) facial palsy, (21) upper limb paralysis, and (22) lower limb paralysis.

To develop the ML models, we excluded the variables of unilateral spatial neglect, smoking status, and history of cerebral infarction. Although we used these variables in the previous model [9], this was done because unilateral spatial neglect was reportedly useful in detecting LVO [13]. However, 5% of LVO cases were judged positive by EMS in the validation cohort of a previous report [9]. Therefore, we considered it difficult for EMS to obtain unilateral spatial neglect. Smoking status and history of cerebral infarction were also excluded because those were judged to be “null” when the patients were unconscious without family members. Thus, all 19 variables could be easily obtained by EMS even if patients were unconscious, and the missing could not be assumed. Finally, 19 variables were used to develop the ML models.

### Definition of Outcomes

All patients were immediately assessed using either CT or MRI at the centers by a neurosurgeon or neurologist and diagnosed with LVO, ICH, SAH, or CI other than LVO. If patients did not have any of these strokes or were diagnosed with conditions other than stroke were considered to have no stroke. LVO was defined as occlusion of the cerebral large vessel, detected by CT arteriography (CTA), MR angiography (MRA), or cerebral angiography, with a low-density area detected with CT or a high-intensity area detected with diffusion-weighted MRI. ICH was defined as a high-density area on CT or a high-intensity area on MRI T1 weighted images of the brain parenchyma. SAH was defined as a high-density area on CT or a high-intensity area on MRI with fluid-attenuated inversion recovery in the subarachnoid space. ICH with SAH accompanied by rupture of the cerebral aneurysm was classified as SAH. CI was defined as a high-intensity area detected by diffusion-weighted MRI, with no occlusion of the cerebral large vessel. Transient ischemic attacks were categorized as no stroke. The definitions of these outcomes were fixed prior to patient enrolment.

### Development of ML Models

To develop the ML models, we used the training cohort for model training and the test cohort for model testing. All variables were categorized, except for age which was treated as continuous without normalization. We selected three different algorithms for developing the models: (1) logistic regression, (2) random forests [14], and (3) extreme gradient descent boosting (XGBoost) [15]. For each algorithm, a softmax function was used to calculate the probability of each type of stroke or no stroke as such that the total probability for each type became 100%. To reduce the risk of misclassification of patients with any type of stroke into the no stroke group, those with a probability of no stroke > 50% were defined as having no stroke. Whereas when the probability of no stroke was < 50%, the stroke type LVO, ICH, SAH, and CI with the highest probability were considered the predicted outcome.

To train the ML models, we used the training cohort, and we performed a grid search and stratified fivefold cross-validation to extract the optimal parameters and check the performance of generalization. The accuracy of the entire model was used as an index to extract the parameters of the model. We then trained the models using the entire training cohort. We estimated the feature importance for random forests and XGBoost. We calculated the relative weights of the beta estimates of each variable in the logistic regression model and presented them as feature importance. After model training was completed, we tested the models to ensure their performance using the test cohort.

### Statistical Analyses

To describe the cohorts, we presented categorical variables as number and percentage and continuous variables as mean and standard deviation. Comparisons between the training and test cohorts were conducted using the Chi-squared test for categorical variables and *t* test for continuous variables.

To evaluate the performance of the models, we calculated the accuracy, sensitivity, specificity, positive predictive value, *F* score, and area under the receiver operating characteristic curve (AUC) for each type of stroke in the training and test cohorts individually. The performance measure for the training cohort was based on the stratified fivefold cross-validation. The definition of the *F* score was as follows:

$$F \mathrm{score}=2\times \mathrm{positive predictive value}\times \mathrm{sensitivity}/\left(\mathrm{positive predictive value}+\mathrm{sensitivity}\right)$$

We also examined the probabilities calculated using the models and the actual probabilities in the test cohort.

We calculated the AUCs of previous scales: GAI2AA [7], Cincinnati Prehospital Stroke Severity scale (CPSSS) [4], Prehospital Acute Stroke Severity scale (PASS) [5], Emergent Large Vessel Occlusion screen (ELVO) [6], JUST score [9], and JUST-7 score [10] for comparison with ML models in the test cohort. As an exploratory analysis, we conducted DeLong test for comparisons between JUST score and the ML models.

All analyses were conducted using open-source Python (version 3.8.0; Python Software Foundation, Beaverton, OR, USA) and JMP 14.0 (SAS Institute Inc., Cary, NC, USA). Two-tailed *p* values of < 0.05 were considered statistically significant.

## Results

### Development of ML Models

**Fig. 1:**
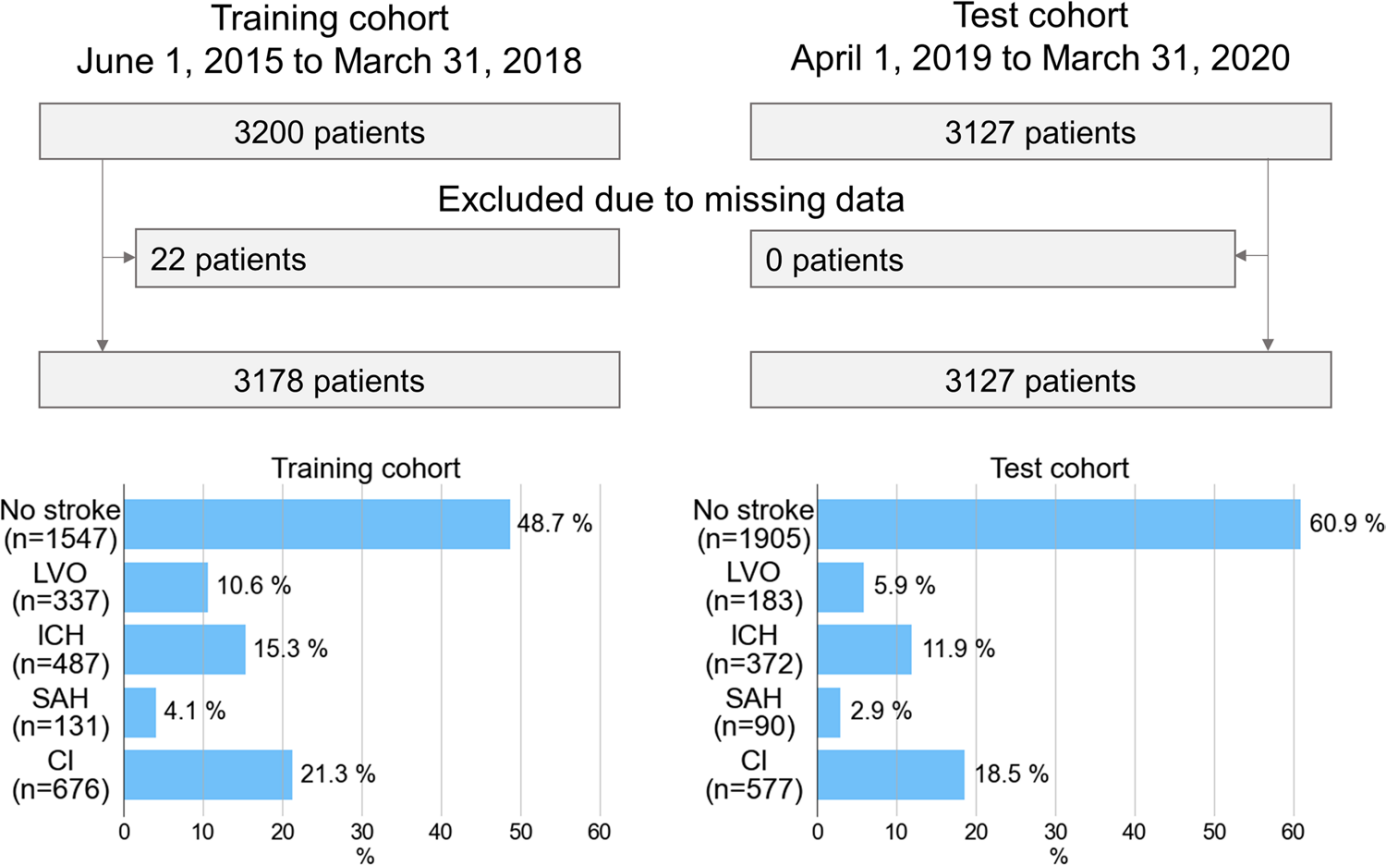
Study flowchart. LVO, large vessel occlusion; ICH, intracranial hemorrhage; SAH, subarachnoid hemorrhage; CI, cerebral infarction other than large vessel occlusion

A total of 3200 patients were initially recruited in the training cohort and 3178 patients were finally included in the analysis, after excluding 22 patients without data on blood pressure (Fig. 1). As a result, there were no missing variables for all 19 variables in the training cohort. The mean age was 71 years, and 53.8% of the patients were men (Table 1). The frequencies of predictive variables ranged from 5% (convulsion) to 55.4% (sudden onset). The final diagnoses were LVO in 337 patients (10.6%), ICH in 487 patients (15.3%), SAH in 131 patients (4.1%), and CI in 676 patients (21.3%) (Fig. 1). Among those suspected of having stroke by the EMS, 1547 (48.7%) did not have a stroke.

**Table 1:** Patients characteristics in the training and test cohorts

Variables	Training cohort (n = 3178)	Test cohort (n = 3127)	*p* value
Male, *n* (%)	1711 (53.8)	1696 (54.2)	0.75
Age-years, mean (SD)	71 (15.4)	70 (17.4)	0.008
Systolic blood pressure > = 165 mmHg, *n* (%)	1419 (44.7)	1196 (38.2)	< 0.001
Diastolic blood pressure > = 95 mmHg, *n* (%)	909 (28.6)	944 (30.2)	0.17
Arrhythmia, *n* (%)	607 (19.1)	460 (14.7)	< 0.001
Conjugate deviation, *n* (%)	429 (13.5)	259 (8.3)	< 0.001
Aphasia, *n* (%)	458 (14.4)	293 (9.4)	< 0.001
Headache, *n* (%)	490 (15.4)	809 (25.9)	< 0.001
Convulsion, *n* (%)	158 (5.0)	126 (4.0)	0.07
Dysarthria, *n* (%)	950 (29.9)	695 (22.2)	< 0.001
Dizziness, *n* (%)	429 (13.5)	575 (18.4)	< 0.001
Nausea or vomiting, *n* (%)	611 (19.2)	864 (27.6)	< 0.001
Sudden onset, *n* (%)	1762 (55.4)	1802 (57.6)	0.08
Symptoms improved after onset, *n* (%)	404 (12.7)	345 (11.0)	0.04
Symptoms progressed after onset, *n* (%)	420 (13.2)	487 (15.6)	0.008
Disturbance of consciousness, *n* (%)	1151 (36.2)	902 (28.8)	< 0.001
Facial palsy, *n* (%)	633 (19.9)	432 (13.8)	< 0.001
Paralysis of upper limbs, *n* (%)	1239 (39.0)	968 (31.0)	< 0.001
Paralysis of lower limbs, *n* (%)	1099 (34.6)	831 (26.6)	< .001

*SD*, standard deviation

**Fig. 2:**
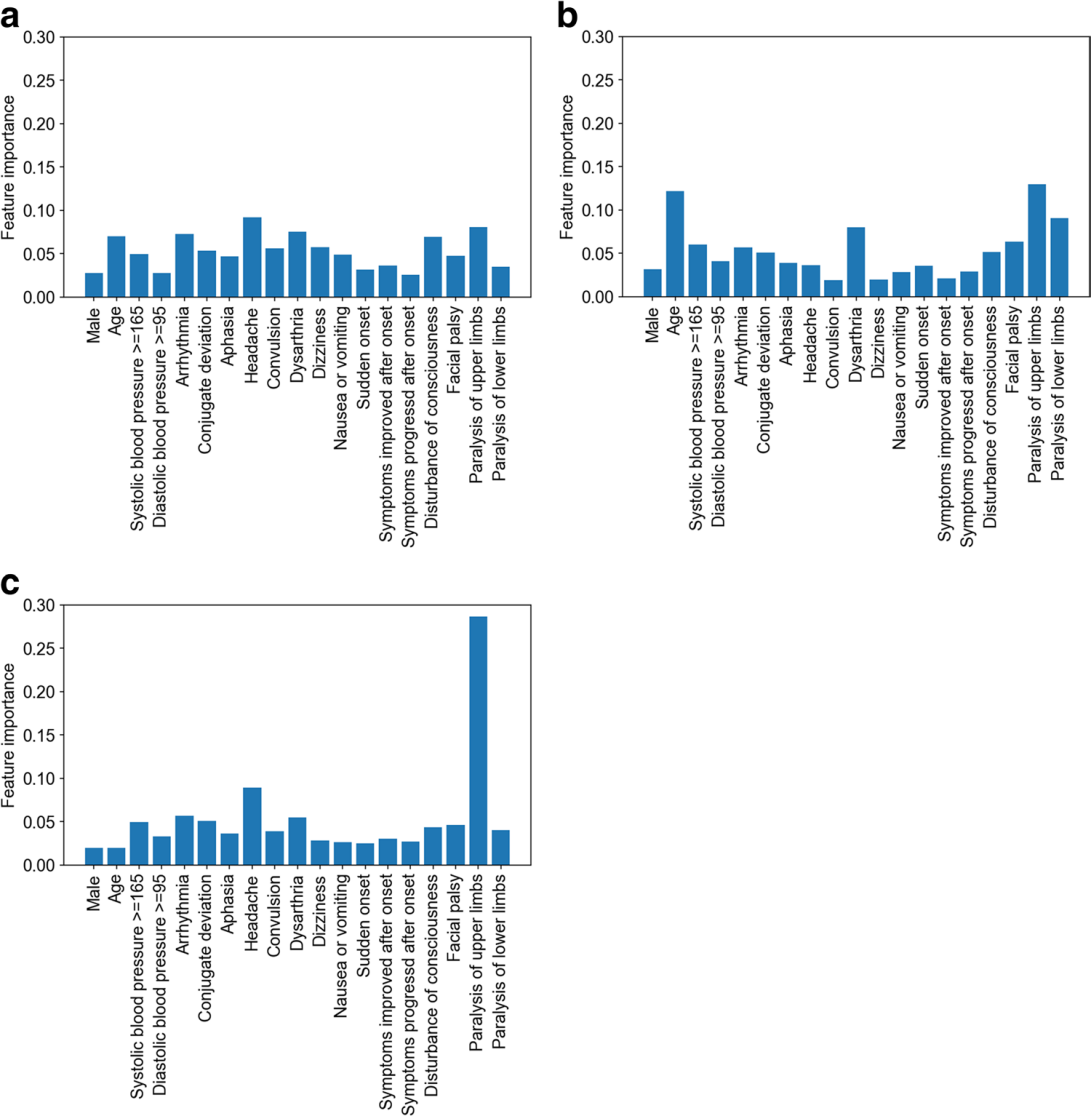
Feature importance. **a** Logistic regression. **b** Random forests. **c** XGBoost. XGBoost, extreme gradient descent boosting

The fivefold cross-validation with the fit parameters (Table 2) showed that the accuracy was the highest with XGBoost (0.623), and those of the logistic regression model and random forest were similar (0.615) (Table 3). The feature importance of variables was different among the three ML models (Figs. 2a, b, and c).

**Table 2:** Parameters for machine learning models

Machine learning models	Parameters
Logistic regression	penalty: l2, C: 0.1, Solver: lbfgs
Random forests	max_depth: 9, max_feature: 3, n_estimators: 1000
XGBoost	learning_rate:0.1, max_depth: 5, min_child_weight: 10, reg_lambda: 0.01, n_estimators: 50

*XGBoost*, extreme gradient descent boosting

**Table 3:** Predictive performance of machine learning models

	Types of stroke	Accuracy	Sensitivity	Specificity	AUC	*F* score	Positive predictive value	Negative predictive value	False negative	False positive
Training cohort										
Logistic regression	No stroke	-	0.8	0.77	0.84	0.73	0.74	0.80	0.20	0.27
	LVO	-	0.49	0.52	0.88	0.95	0.56	0.94	0.51	0.05
	ICH	-	0.43	0.45	0.79	0.91	0.46	0.90	0.57	0.09
	SAH	-	0.33	0.4	0.89	0.99	0.56	0.97	0.67	0.01
	CI	-	0.44	0.44	0.74	0.85	0.44	0.85	0.56	0.15
	Overall	0.615	0.5	0.51	-	0.89	0.55	0.89	0.50	0.11
Random forests	No stroke	-	0.79	0.77	0.85	0.76	0.76	0.79	0.21	0.24
	LVO	-	0.51	0.5	0.88	0.94	0.51	0.94	0.49	0.06
	ICH	-	0.42	0.45	0.79	0.92	0.5	0.90	0.58	0.08
	SAH	-	0.31	0.41	0.91	0.99	0.64	0.97	0.69	0.01
	CI	-	0.48	0.45	0.75	0.82	0.42	0.85	0.52	0.18
	Overall	0.615	0.5	0.52	-	0.89	0.57	0.89	0.50	0.11
XGBoost	No stroke	-	0.8	0.78	0.85	0.76	0.76	0.80	0.20	0.24
	LVO	-	0.51	0.52	0.88	0.95	0.53	0.94	0.49	0.05
	ICH	-	0.43	0.45	0.78	0.91	0.48	0.90	0.57	0.09
	SAH	-	0.37	0.42	0.92	0.98	0.5	0.97	0.63	0.02
	CI	-	0.48	0.47	0.75	0.85	0.46	0.86	0.52	0.15
	Overall	0.623	0.52	0.53	-	0.89	0.55	0.89	0.48	0.11
Test cohort										
Logistic regression	No stroke	-	0.8	0.71	0.84	0.8	0.81	0.69	0.20	0.29
	LVO	-	0.34	0.97	0.89	0.36	0.39	0.96	0.66	0.03
	ICH	-	0.43	0.92	0.82	0.42	0.42	0.92	0.57	0.08
	SAH	-	0.27	0.97	0.87	0.25	0.23	0.98	0.73	0.03
	CI	-	0.44	0.86	0.78	0.43	0.42	0.87	0.56	0.14
	Overall	0.65	0.46	0.89	-	0.45	0.46	0.89	0.54	0.11
Random forests	No stroke	-	0.8	0.74	0.84	0.81	0.83	0.70	0.21	0.26
	LVO	-	0.39	0.96	0.89	0.39	0.39	0.96	0.61	0.04
	ICH	-	0.41	0.94	0.82	0.43	0.46	0.92	0.59	0.06
	SAH	-	0.16	0.98	0.85	0.18	0.23	0.98	0.84	0.02
	CI	-	0.51	0.83	0.78	0.45	0.41	0.88	0.49	0.17
	Overall	0.65	0.46	0.89	-	0.45	0.45	0.89	0.55	0.11
XGBoost	No stroke	-	0.79	0.74	0.84	0.81	0.83	0.69	0.21	0.26
	LVO	-	0.43	0.96	0.88	0.41	0.4	0.96	0.57	0.04
	ICH	-	0.4	0.92	0.81	0.4	0.41	0.92	0.60	0.08
	SAH	-	0.23	0.97	0.86	0.22	0.21	0.98	0.77	0.03
	CI	-	0.48	0.85	0.78	0.45	0.42	0.88	0.52	0.15
	Overall	0.65	0.47	0.89	-	0.46	0.45	0.89	0.53	0.11

*AUC*, area under the receiver operating characteristic curve; *LVO*, large vessel occlusion; *ICH*, intracranial hemorrhage; *SAH*, subarachnoid hemorrhage; *CI*, cerebral infarction other than large vessel occlusion; *XGBoost*, extreme gradient descent boosting

### Testing the ML Models

In the test cohort, there were 3127 patients without missing data (Fig. 1). Although the age and sex distributions were generally similar between the training and test cohorts, the frequencies of the predictive variables were different between the two cohorts (Table 1). The final diagnoses were LVO in 183 patients (5.9%), ICH in 372 patients (11.9%), SAH in 90 patients (2.9%), and CI in 577 patients (18.5%) (Fig. 1). Finally, there were 1905 patients (60.9%) without stroke.

**Fig. 3:**
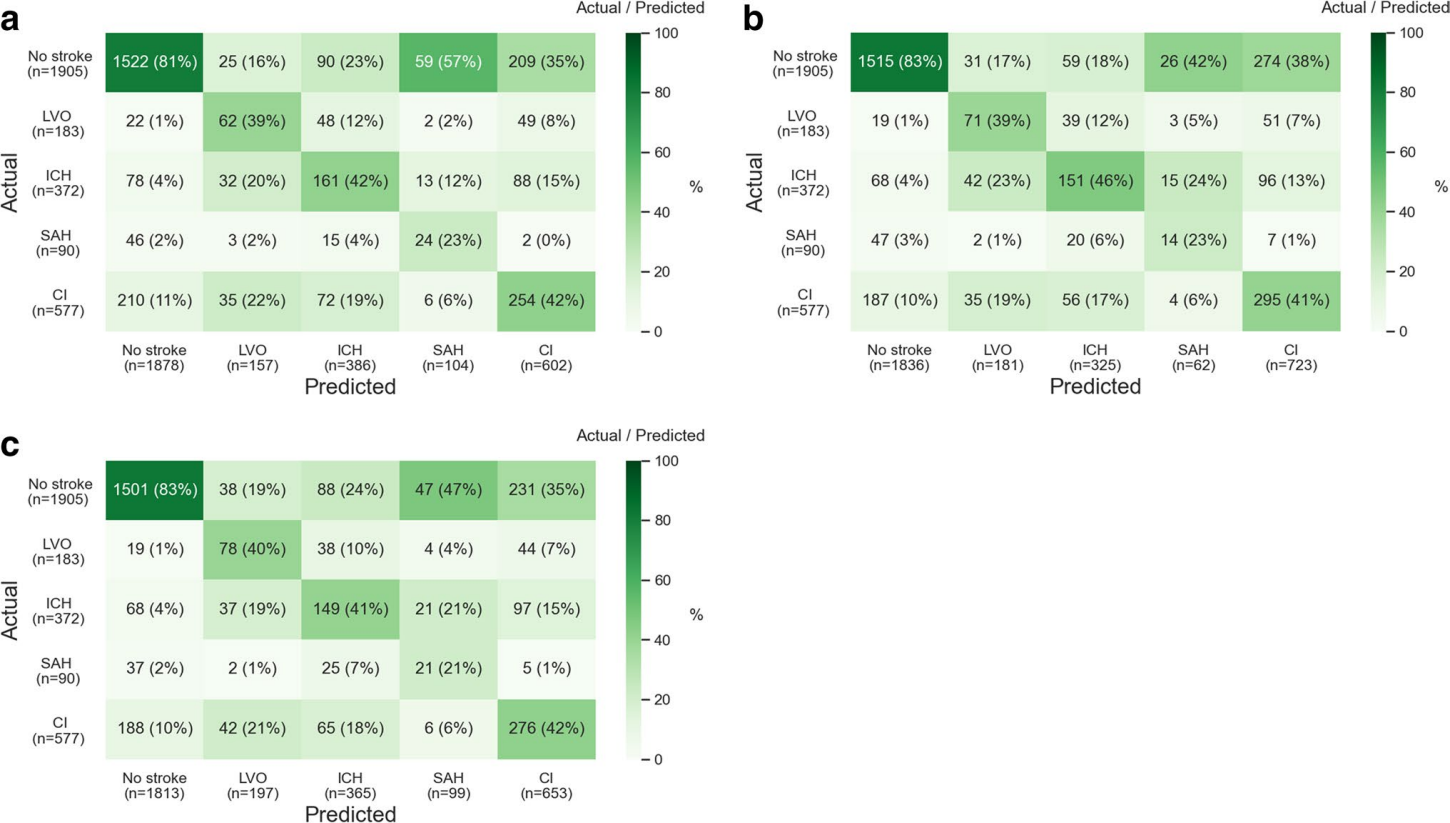
Classification ability. **a** Logistic regression. **b** Random forests. **c** XGBoost. LVO, large vessel occlusion; ICH, intracranial hemorrhage; SAH, subarachnoid hemorrhage; CI, cerebral infarction other than large vessel occlusion; XGBoost, extreme gradient descent boosting

**Fig. 4:**
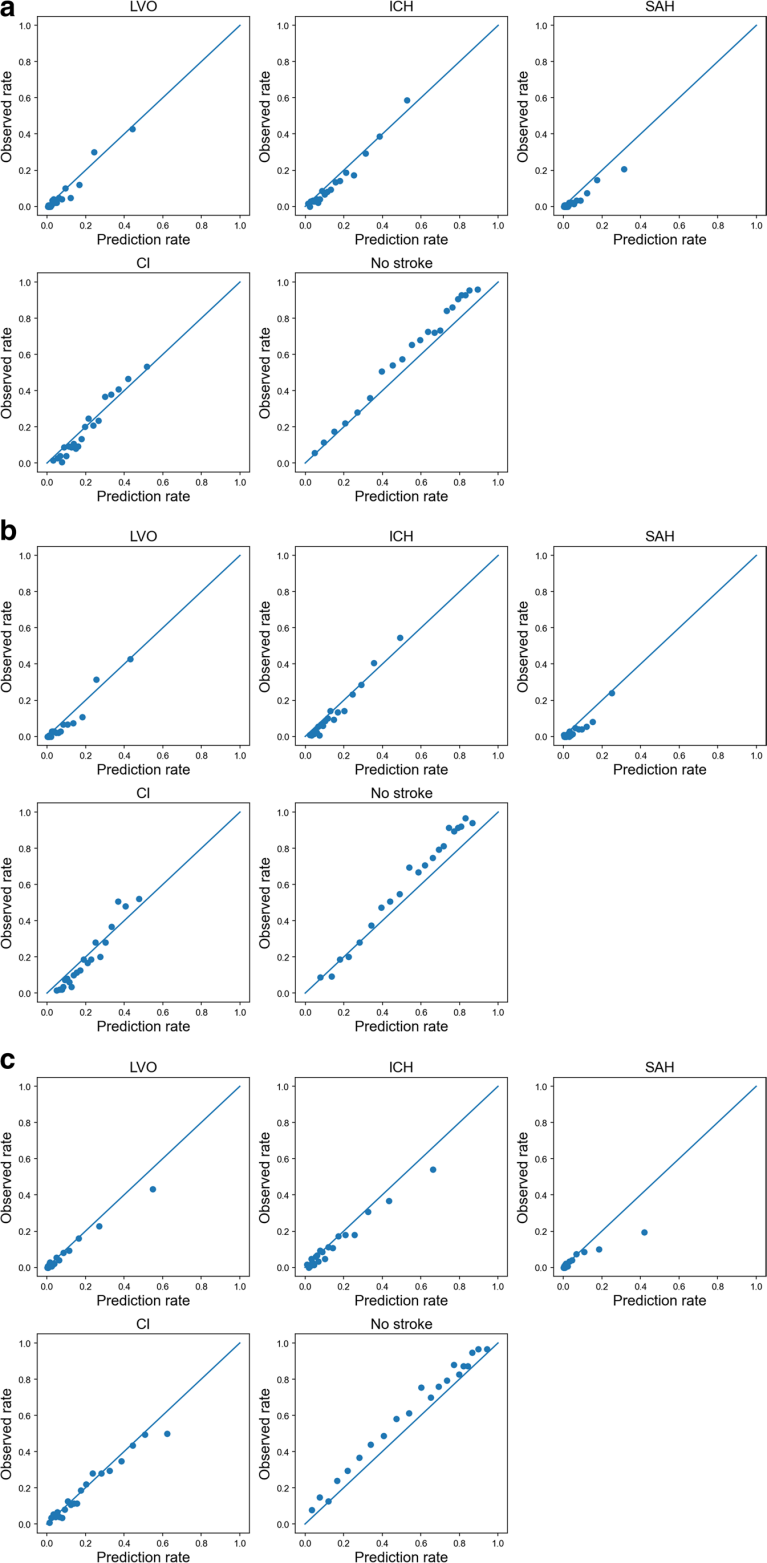
Calibration of machine learning models. **a** Logistic regression. **b** Random forests. **c** XGBoost. LVO, large vessel occlusion; ICH, intracranial hemorrhage; SAH, subarachnoid hemorrhage; CI, cerebral infarction other than large vessel occlusion; XGBoost, extreme gradient descent boosting

The overall accuracies were 0.65 for all ML models and the positive predictive values, sensitivities, specificities, AUCs, and *F* scores were stable in the test cohort (Table 3). The classification abilities were generally fair for all ML models (Figs. 3a, b, and c). The misclassifications for the prediction of no stroke among 183 patients with actual LVO were 22, 19, and 19 with logistic regression, random forests, and XGBoost, respectively (Figs. 3a, b, and c). The predicted probabilities of the four types of stroke and no stroke were also generally fair for all ML models (Figs. 4a, b, and c).

### Comparisons of Prediction of LVO

**Fig. 5:**
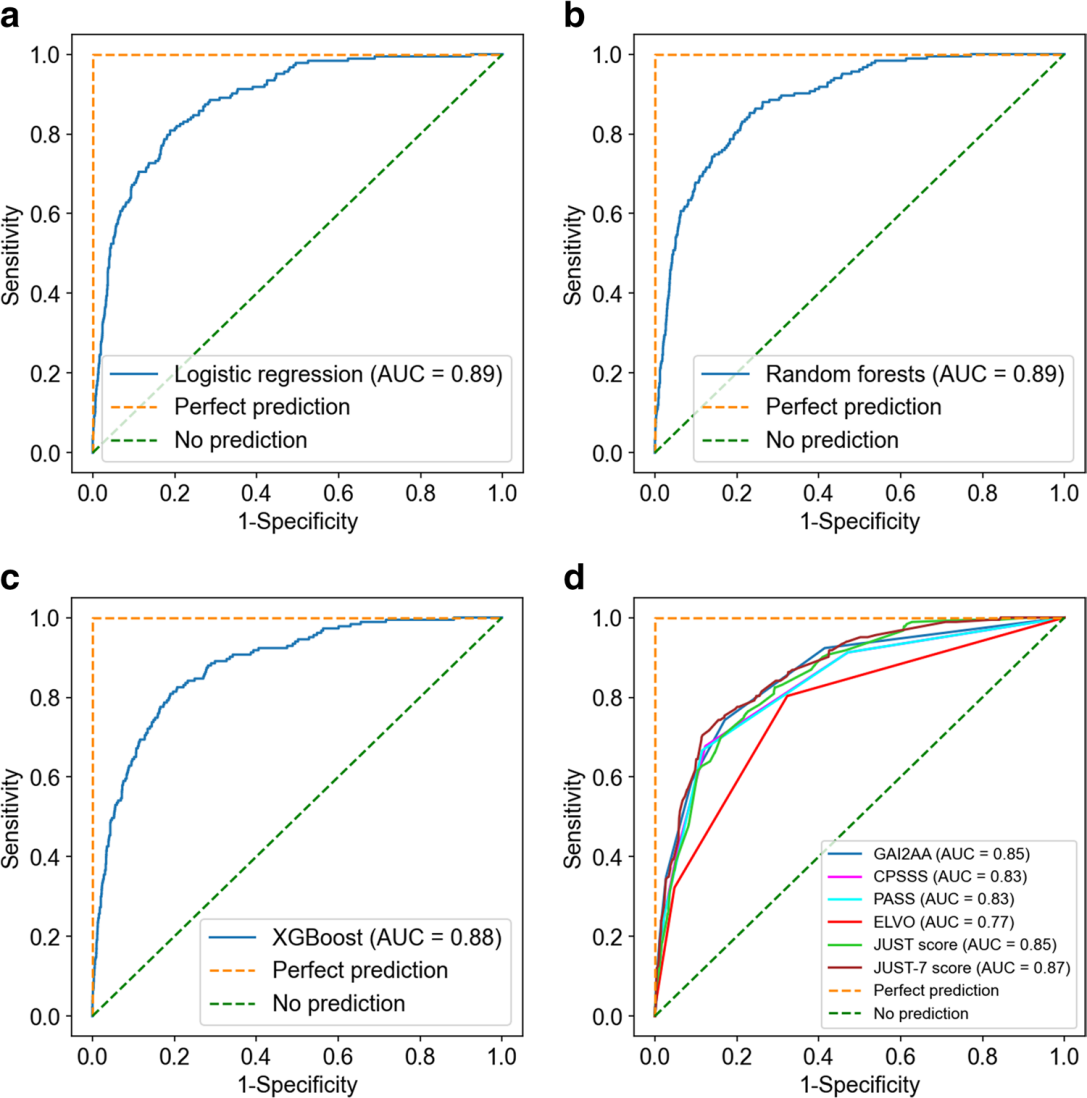
AUCs of machine learning models and previous scales for large vessel occlusion. **a** Logistic regression. **b** Random forests. **c** XGBoost. **d** Previous scales. AUC, area under the receiver operating characteristic curve; CPSSS, Cincinnati Prehospital Stroke Severity scale; ELVO, Emergent Large Vessel Occlusion screen; JUST, Japan Urgent Stroke Triage; JUST-7, 7-Item Japan Urgent Stroke Triage; PASS, Prehospital Acute Stroke Severity scale; XGBoost, extreme gradient descent boosting

The AUCs for LVO in logistic regression, random forests, and XGBoost were 0.89, 0.89, and 0.88, respectively, in the test cohort (Figs. 5a, b, and c). The other scales had similar AUCs, around 0.83–0.87, other than ELVO that had an AUC of 0.77 (Fig. 5d). Except for JUST and JUST-7 scores, which were previous versions of the ML models, the highest positive predictive value was GAI2AA (29%), while it was 36% for JUST-7 score and 39–40% for the three ML models (Table 4). The AUCs of the ML models were not significantly different from the JUST score (*p* = 0.13 for logistic regression; *p* = 0.12 for random forests; *p* = 0.21 for XGBoost).

**Table 4:** Predictive abilities of LVO scales

Previous LVO scales	Positive predictive value	Sensitivity	Specificity	AUC	*F* score
GAI2AA	0.29	0.59	0.91	0.85 [0.82–0.89]	0.39
CPSSS	0.25	0.68	0.88	0.83 [0.80–0.87]	0.37
PASS	0.26	0.67	0.88	0.83 [0.80–0.87]	0.38
ELVO	0.13	0.8	0.68	0.77 [0.73–0.81]	0.23
JUST score	0.34	0.26	0.97	0.85 [0.81–0.88]	0.29
JUST-7 score	0.36	0.39	0.96	0.87 [0.83–0.90]	0.38
Logistic regression	0.39	0.34	0.97	0.89 [0.85–0.92]	0.36
Fandom forests	0.39	0.39	0.96	0.89 [0.85–0.91]	0.39
XGBoost	0.40	0.43	0.96	0.88 [0.85–0.91]	0.41

*AUC*, area under the receiver operating characteristic curve; *CPSSS*, Cincinnati Prehospital Stroke Severity scale; *ELVO*, Emergent Large Vessel Occlusion screen; *JUST*, Japan Urgent Stroke Triage; *JUST*-*7*, 7-Item Japan Urgent Stroke Triage; LVO, large vessel occlusion; *PASS*, Prehospital Acute Stroke Severity scale; *XGBoost*, extreme gradient descent boosting

## Discussion

We applied ML methods to develop prediction models for calculating the predicted probabilities of each type of stroke and suggested the most likely type of stroke at a prehospital stage. This study is the first to use ML methods applied in clinical prediction models for patients suspected of having an acute stroke. Although JUST and JUST-7 scores had excellent predictive abilities to differentiate patients suspected of having an acute stroke, they were operationally complex as they calculated the probabilities for each type of stroke separately and judged stroke with higher priority. The three different algorithms had similar accuracy among logistic regression, random forests, and XGBoost, although the feature importance of the variables used differed. The AUCs of the ML models were satisfactory (0.88–0.89) and higher than those of previous models.

Previous prediction scales could only classify whether a patient had an LVO or did not have one. In addition, those were not satisfied with discrimination abilities because of the necessity of a cut-off value. Precision was reported in 29% of patients with GAI2AA [7], 25% with CPSSS [4], 26% with PASS [5], and 13% with ELVO [6]. On the other hand, the ML models had higher positive predictive values (39% in the logistic regression, 39% in the random forest, and 40% in the XGBoost) than previous scales. Moreover, among 183 LVO cases, only 22 cases were finally classified as not stroke by logistic regression, 19 cases by random forest, and 19 cases by XGBoost. This suggests that LVO is less likely to be missed while maintaining high precision of LVO. The relatively lower sensitivity of the ML models for LVO should be carefully interpreted. Although the sensitivity and specificity were always a trade-off, the ML models, as well as JUST and JUST-7 scores, discriminate 4 types of strokes. Therefore, some LVOs with acute neurological signs could be inevitably classified into other types of strokes and the sensitivity would be decreased. However, such patients were generally classified into LVO when only two outcomes (LVO vs no LVO) were predicted.

We have already distributed the application of JUST score on mobile devices and web browsers, and many EMS currently utilize this score to transport patients suspected of having acute stroke in Japan. By using such an application, EMS or other physicians who encounter patients suspected of having acute stroke can easily predict probabilities and the type of stroke, and efficiently transport patients to the capable facilities. ML has been applied in various fields owing to its high predictive performance. Applying ML methods improves predictive ability compared with conventional predictive tools [11, 12]. Because previous conventional predictive tools used integers of the importance of variables, it could be possible to improve the predictive ability without converting variables to integers. However, our study showed the ML models did not dramatically improve the predictive abilities than the previous version of the JUST score because we used the same potential predictors. Even so, the performance of our ML models could be improved by accumulating data in real-time. Another major advantage of using ML is the ease of processing a large amount of information within a short period of time.

We used basic ML algorithms to develop these models, and other predictive tools using artificial intelligence (AI) could also be applicable with other potential variables and such systems could refine prediction models based on any potential data. Such an AI-based prehospital stroke prediction model should be implemented in the future as the ultimate version of the JUST score. The use of the AI-based prehospital stroke prediction model could be extremely significant in low-resource settings where the transportation system is not well organized or imaging diagnostic assessments are available at limited facilities. If precise triage could be achieved using small mobile devices, such use could help patients suspected of having an acute stroke to be transported to capable facilities where imaging studies are available without unnecessary transportations [16].

This study had several limitations. First, the models in this study utilized binary categorical data from previous studies, except for age, to construct the features. Therefore, the predictive abilities of ML models can be penalized. Although the development of an ML model with higher performance should be a challenge for future studies, the current ML models could provide the lowest performance, and these findings should be considered as the minimum. Second, the model utilizes 19 variables for prediction. In an emergency setting, a prediction model with fewer variables should be built because many patients, at a prehospital stage, would have a variety of conditions other than stroke. Thus, our previous attempt with a shorter version of the JUST score (JUST-7) should be incorporated in developing AI-based models. Third, the predictive abilities depend on the prevalence of the target conditions. If the prevalence of no stroke was substantially high, the utility of JUST-ML would be deteriorated. Therefore, any prediction tools should be carefully interpreted in conjunction with the circumstance where the tool was used. Finally, the predicted probabilities of SAH and no stroke were slightly deviated from the perfect fit. These differences should be considered the differences in the cohorts. Because this study was conducted in a local area in Japan, the generalizability of the JUST-ML should be attested in other settings. The most attractive abilities of the ML-based model are obtaining local data and refining the models based on these data. The application of JUST-ML should be investigated globally.

## Conclusions

We have developed an ML model (JUST-ML) that simultaneously predicts the type of stroke at a pre-hospital stage with high accuracy, which could assist EMS or primary care providers with triaging patients suspected of having an acute stroke.

## Data Availability

The data that support the findings of this study are available from the corresponding author upon reasonable request.

## Code Availability

The code used to generate results shown in this study is available from the corresponding author upon request.
